# Algo's Integrated Knowledge Translation Process in Homecare Services: A Cross-Sectional Correlational Study for Identifying its Level of Utilization and its Associated Characteristics

**DOI:** 10.1177/00084174211064495

**Published:** 2021-12-14

**Authors:** Mélanie Ruest, Guillaume Léonard, Aliki Thomas, Johanne Desrosiers, Manon Guay

**Keywords:** Home care services, Integrated knowledge translation, Occupational therapy, Approche d’application des connaissances intégrée, ergothérapie, services de soutien à domicile

## Abstract

**Background.** Algo is an integrated knowledge translation (IKT)-based algorithm for supporting occupational therapists (OTs) with skill mix for selecting bathing equipment. While IKT approaches are increasingly valued in implementation science, their benefits with respect to the utilization of knowledge in clinical settings are scarcely documented. **Purpose.** To identify Algo's level of utilization and the characteristics associated with its level of utilization. **Method.** A cross-sectional correlational study was conducted with OTs working in homecare services (HCS) through an online survey based on Knott and Wildavsky's classification and the *Promoting Action on Research Implementation in Health Services* (*PARIHS*) framework. **Findings.** Almost half (48%) of the OTs surveyed (n = 125; participation rate: 16%) reached one of the seven levels of utilization. While *Evidence* characteristics are perceived as facilitators to its utilization, *Context* statements indicate an unfavorable organizational climate to the implementation of change. **Implications.** Strategies should target additional stakeholders (e.g., HCS managers) and organizational adjustments in HCS to sustain Algo's utilization.

## Résumé

**Description.** L’Algo est un algorithme basé sur une approche d’application des connaissances (AC) intégrée qui aide les ergothérapeutes à sélectionner l’équipement de soins d’hygiène en recourant au chevauchement des compétences. Si les approches d’AC intégrée sont de plus en plus valorisées dans le domaine de l’application des connaissances, leurs bénéfices quant à l’utilisation des connaissances en contexte clinique sont peu documentés. **But.** Déterminer le niveau d’utilisation de l’Algo et les caractéristiques qui y sont associées. **Méthodologie.** Une étude corrélationnelle transversale a été réalisée auprès d’ergothérapeutes travaillant dans les services de soutien à domicile par l’entremise d’un sondage en ligne basé sur la classification de Knott et Wildavsky et sur le cadre conceptuel PARIHS. **Résultats.** Près de la moitié, soit 48% des ergothérapeutes sondés (n = 125; taux de participation : 16%), ont atteint l’un des sept stades d’utilisation. Si les caractéristiques de l’outil sont perçues comme facilitant son utilisation, les énoncés relatifs au contexte indiquent un climat organisationnel défavorable à la mise en œuvre du changement. **Conséquences.** Les stratégies doivent cibler d’autres parties prenantes (p. ex., les gestionnaires des services de soutien à domicile) et viser des changements organisationnels dans les services de soutien à domicile afin de favoriser l’utilisation de l’Algo.

## Background

In Canada, health care is a responsibility allocated to the different provinces through different programs (e.g., homecare services [HCS]). Skill mix (i.e., cross-skilling of human resources with extended roles [Stanmore and Waterman, 2007]) is used to accelerate the allocation and the delivery of services to the population (Smith et al., 2018). In homecare occupational therapy (OT), nonOTs (e.g., home health aides) can participate in the selection of bathing equipment (e.g., grab bar) for older adults living at home and meeting the criteria for a “simple clinical situation” (Guay et al., 2012). In Quebec (Canada), the organization responsible to regulate the professional exercise of OT (i.e., *Ordre des ergothérapeutes du Québec* [*OEQ*]) emphasizes the importance of making “tools” available to nonOTs who participate in the selection process of bathing equipment to support the rigorous utilization of skill mix (Ordre des ergothérapeutes du Québec, 2005, 2008).

For this reason, an evidence-based clinical algorithm called “Algo” has been deployed in Quebec HCS to support OTs and nonOTs in this skill mix process since 2013 (Guay et al., 2014). Algo was first developed between 2009 and 2012 using an integrated knowledge translation (IKT) approach to optimize its implementation in clinical settings (Guay et al., 2014; Guay et al., 2019). In the field of knowledge translation, collaboration between researchers and knowledge users (e.g., health care professionals) in the co-production of knowledge is increasingly valued as it can contribute toward reducing documented gaps between scientific knowledge and its utilization by health professionals in clinical settings (Jull et al., 2017; Kothari & Wathen, 2013). According to the Canadian Institutes of Health Research principles for knowledge translation (KT), researchers and potential knowledge users should (a) decide on the formulation of the research questions, (b) interpret the results of the study, and (c) participate in the preparation and dissemination of main messages about the results and work toward moving these into practice (Parry et al., 2015). Despite the potential benefits of IKT approaches, little is known about the concrete impact of adhering to these principles on the dissemination and uptake of knowledge in health care systems (Gagliardi et al., 2016; Kreindler, 2018). As an IKT-based clinical algorithm, the identification of Algo's level of utilization in HCS, following its development and its dissemination in collaboration with end users, offers a realist perspective to evaluate the clinical benefits related to the use of IKT approaches.

Following the development of Algo using IKT principles 1 and 2 (decide on the formulation of the research questions and interpret the results of the study; Guay et al., 2014), four passive (i.e., reference manual for OTs, user guide for nonOTs, training plan and website) and one active (i.e., training offered in the workplace) strategies were developed in collaboration with OTs and nonOTs (Guay et al., 2013) to support its utilization. Though the facilitation strategies developed for Algo were triangulated with results from KT systematic reviews on interventions most often used in rehabilitation (Jones et al., 2015), the benefits of its IKT characteristics in HCS and OT practice are still unknown. As the implementation of knowledge involves a unique interplay of individual and organizational characteristics, conceptual frameworks are recognized to support the identification of barriers and enablers as well as the development of effective facilitation strategies (Colquhoun et al., 2010; Tabak et al., 2012).

### Theoretical Frameworks

Two conceptual frameworks and one classification were used to inform Algo's IKT process. The first framework “*Promoting Action on Research Implementation in Health Services* (*PARIHS*)” documents the process by analyzing the interrelation of three dimensions: *Evidence*, *Context*, and *Facilitation* (Kitson et al., 1998). The *PARIHS* framework has extensive literature on its development (Kitson et al., 1998, 2008; Stetler et al., 2011) and its usability in clinical settings to investigate the utilization of evidence-based knowledge (Gibb, 2013; Hutchinson et al., 2012; McKillop et al., 2012). The *Evidence* dimension (i.e., characteristics of knowledge) was documented by considering characteristics valued in general and those specifically related to Algo. The *Context* dimension (i.e., environment of implementation) was represented by the Quebec HCS of the health care system, where OTs are invited to use Algo. Finally, the *Facilitation* dimension (i.e., implementation strategies), was represented by the interventions described above.

Selected to complement the characteristics illustrated with the matrix form of the *PARIHS* framework, the *Consolidated Framework for Implementation Research* (*CFIR*) guides the implementation process through five main components: *Intervention Characteristics*, *Outer Setting*, *Inner Setting*, *Characteristics of Individuals* and *Process* (Damschroder et al., 2009). In this study, the *CFIR* allowed to further identify some characteristics related to the end users and the context to consider for Algo's IKT process.

Finally, the 7-stage classification of Knott and Wildavsky (Knott & Wildavsky, 1980) was used to operationalize Algo's levels of utilization. This classification conceptualizes the progression of knowledge utilization through a chain of cumulative stages in that each one must be reached prior to moving to the next: (1) reception (i.e., Has the knowledge been presented to you?); (2) cognition (i.e., Have you consulted the knowledge?); (3) reference (i.e., Has the knowledge influenced your decisions?); (4) effort (i.e., Have you made an effort to adopt the knowledge?); (5) adoption (i.e., Do you use the knowledge?); (6) implementation (i.e., Is the knowledge integrated into the policies and the procedures of the setting?); and (7) impact (i.e., Has the knowledge had a positive effect on the practices?). Empirical studies based on this classification supported the use of the proposed conceptualization (Davidson & Nowicki, 2012; Siu et al., 2009).

### Objectives

The purpose of this study was to identify (1) the level of utilization of Algo and (2) the characteristics (related to knowledge, end users, and context) associated with a higher level of utilization, 2 years after its diffusion to analyze Algo's IKT process in HCS of the health care system.

## Methods

### Design

This was a cross-sectional correlational study based on an embedded concurrent mixed methods design (QUAN [qual]); Creswell, 2014). The quantitative component (QUAN) allowed to identify Algo's level of utilization and to initiate the documentation of its associated characteristics. The qualitative (qual) component consisted of comments about (1) the level of utilization (for each stage of the classification of Knott and Wildavsky) and (2) the level of agreement or disagreement (for each characteristic documented according to the frameworks) identified in the quantitative component. Finally, the results of the components were integrated into a complementary approach to deepen the characteristics related to Algo's IKT process.

### Participants and Recruitment

The target population was OTs members of the *OEQ* who had previously indicated that they worked in HCS. To be eligible, OTs had to be able to read and answer questions in French or English. Using a convenience sampling method (no exclusion criteria), an invitation to complete an online survey (*LimeSurvey* platform) was distributed by e-mail to OTs that had previously accepted to be contacted for research purposes.

### Variables

**Dependent variable.** The level of utilization of Algo was measured with an adapted version of the 7-stage classification of Knott and Wildavsky (1980). The content of questions referring to stages 5 (adoption) and 6 (implementation) was interchanged to better correspond to the clinical reality of knowledge process use as the utilization of knowledge often precedes its integration in the policies and procedures of a clinical setting.

**Independent variables.** Based on the *PARIHS* and *CFIR* frameworks, the characteristics related to Algo's IKT process were measured and were regrouped into three themes: (1) the knowledge (i.e., Algo); (2) the context (i.e., HCS and health care system including the end users); and (3) the facilitation (e.g., implementation strategies).

### Instrument

**Development of the survey.** The first author (MR) developed a preliminary version of the survey in French, based on (1) the literature related to Algo's development, (2) the principles of Dillman (Dillman et al., 2009), and (3) the *PARIHS* guide used to facilitate its operationalization (Stetler et al., 2011). Except for the sociodemographic questions, each survey question or statement referred to a level of utilization of Algo or a characteristic based on the selected frameworks. The survey was reviewed by five experts (KT field and Algo's development), from three universities in Quebec, and two OTs. To document variables not covered by the *PARIHS* framework, additional statements related to the *Outer Setting* and *Intervention* *Characteristics *of the *CFIR* framework were added to the survey (Damschroder et al., 2009). A pretest was performed by the first author (MR) with HCS stakeholders (n = 9; seven OTs and two managers) in their workplace. Principles of the *Think aloud* approach (Arocha & Patel, 2008) were used to gather feedback on the survey.

**Final version of the survey.** In total, the survey contained 28 questions and statements. Seven questions, based on the 7-stage classification of Knott and Wildavsky, were designed to document levels of Algo's utilization. The response scale was dichotomous (Yes/No) for stages 1, 6, and 7, and a 4-point Likert scale was used (Never; Rarely; Occasionally; Frequently) for stages 2–5. Twenty-one (21) questions or statements (e.g., Algo is [or could be] a useful source of knowledge to meet the needs of equipment selection for our clients’ hygiene care), referring to characteristics about Algo's utilization and based on the variables (e.g., relative advantage) of the *PARIHS* (eleven questions) and *CFIR* (six questions) frameworks (Table 2), were answered with a 4-point Likert scale (Strongly Disagree; Disagree; Agree; Strongly Agree). Textboxes were provided below each statement to elaborate responses. Four (4) sociodemographic questions were asked to participants about their academic and professional backgrounds.

### Data Collection

The invitation e-mail was sent to eligible participants by the *OEQ* on September 25, 2015 and a reminder e-mail was sent on February 4, 2016. The survey was available in French and English.

### Data Analysis

Quantitative data were analyzed with *Statistical Package for the Social Sciences* (SPSS (2016); version 23, IBM Corp; Armonk, NY, United States). Descriptive statistics were first used to summarize the level of utilization of Algo. Spearman's correlational analyses were conducted to verify the presence of an association between (1) the maximal level of utilization of Algo reached and (2) the characteristics related to its implementation according to the participants of the study. To increase reproducibility, the alpha thresholds of associations were set at *p* ≤ .005 and ≤ .05, respectively, considered as statistically significant and as statistically suggestive (Benjamin et al., 2018). A binary logistic regression model (*p*-value of ≤ .05) was performed with characteristics associated with a higher level of utilization of Algo to investigate their contribution for predicting the outcome of using (or not) Algo through the stages of utilization among OTs reached in this study. Confidence intervals of 95% were calculated for each odds ratio coefficient.

For the qualitative data, the comments were imported from *LimeSurvey* to the NVivo 10 platform (QSR (2016) International Pty Ltd; Australia). Each utterance was coded deductively by the first author (MR) with a coding grid based on the conceptual frameworks. Supplementary variables were added to the coding grid inductively (e.g., use of in-house “tools”). Following the individual analysis of both datasets, results were integrated using mixed methods matrixes (O’Cathain et al., 2010) through a joint display to triangulate the perspectives of participants. For each level of utilization and related characteristic documented, the levels of agreement or disagreement collected (quantitative component) were analyzed in combination with the participants’ feedback (qualitative component) to corroborate or balance the results.

### Ethics Approval and Consent to Participate

This research project has been approved by the Research Ethics Committee of the *Centre intégré universitaire de santé et de services sociaux (CI[U]SSS) de l’Estrie – Centre hospitalier universitaire de Sherbrooke* (MP-22-2016-532). Virtual consent was obtained from the participants prior to data collection.

## Results

### Characteristics of Participants

Among the total 4,886 OTs registered in *OEQ* in 2015, 18% (n = 886) were working in HCS. Among the 787 OTs that accepted to be contacted for research purposes, 470 opened the e-mail invitation. One hundred eighty-seven (187) responded to the survey; 125 provided complete answers (participation rate: 16%) for the quantitative component and 89 provided data for the qualitative component. The participants were from 21 out of 22 *Centres intégrés [universitaires] de santé et de services sociaux* (*CI[U]SSS*; from 15 of the 16 administrative regions of the province of Quebec). The participants completed their OT degree between 1979 and 2015, with the bachelor’s degree being the highest academic level reached for 73% of them. Others had a professional master in OT (n = 24) or a master’s in research (n = 6). Participants had an average of 15 years of professional experience (range of 0.5–36). In HCS, participants had an average of 10 years of professional experience (range of 0.3–29). The average completion time of the survey was 21.7 min (range of 5–187).

### Level of Utilization of Algo

Among the 125 OTs (that provided complete answers), 59 reached one of the seven levels of utilization of Algo. Among the 59 OTs, 11% reported having had the opportunity to be introduced to Algo (stage 1; n = 2) and/or having had read and briefly analyzed the algorithm (stage 2; n = 12). For the intermediate levels of utilization, 12% of participants confirmed having had referred to Algo (stage 3; n = 4) in their professional practice (e.g., clinical reasoning, discussion with colleagues) or made efforts to implement it in their clinical setting (stage 4; n = 11). Finally, for the advanced levels of utilization, 24% of participants reported having had used Algo in their professional practice (stage 5; n = 11), implemented it in the policies and procedures of their clinical setting (stage 6; n = 1), or reported the observation of a beneficial impact for patients in HCS (stage 7; n = 18).

### Characteristics Associated With the Utilization of Algo

Based on *PARIHS* and *CFIR* frameworks, only two variables related to the knowledge were associated with a higher level of utilization of Algo by OTs in HCS: (1) the valorization of scientific knowledge in the professional practice and (2) the relative advantage of Algo (i.e., Algo is viewed as a useful source of knowledge to meet the needs of patients with hygiene care difficulties; see Table 1). The valorization of evidence-based knowledge by OTs (*r *= .26 *p* = .003) showed a statistically significant and moderate association, while the relative advantage of the tool (*r* = .24 *p* = .01) showed a statistically suggestive and moderate association with higher levels of utilization of Algo. The multivariate binary logistic regression revealed that three factors are significantly associated with Algo's utilization. First, the promotion of scientific knowledge in their professional practice seems to increase the odds of having reach one of Algo's seven stages of utilization (OR = 2.517 *p* = .020). The model also revealed that participants who responded “Agree” to the relative advantage of Algo were less likely to have reached one of Algo’s stages of utilization than the participants who responded “Strongly Agree” (OR = 0.296 *p* = .004). Indeed, OTs that strongly perceived a relative advantage of Algo are 4 times more likely to be among the participants who achieved one of the seven levels of utilization. Finally, each supplementary year of professional experience of OTs in HCS favors the odds of having reached one of Algo's levels of utilization (OR = 1.13 [1.03–1.23]). Professional experience (years) of OTs was also tested in the multivariable model but was not statistically significant in the model (*p* > .05). Globally, this model explained 21.5% (Nagelkerke *R^2^*) of the variance related to the progression of Algo through the levels of utilization and correctly classified 67.2% of the cases.

**Table 1.** Associations Between Algo's Levels of Utilization and Characteristics of its IKT Process

Variables	Spearman coefficients (*p*-value)
**Evidence variables** (i.e., Algo)	
*PARIHS* characteristics	
Characteristics of the targeted evidence-based practice *Relative advantage*	0.24*^1^ (.01)
Research and published guidelines *Scientific* *Clinical* *Local opinions*	0.26**^2^ (.003) −0.11 (.22) −0.01 (.89)
Clinical experience Clinical experience in homecare services	0.003 (.97) 0.17 (.06)
Patient experiences, needs, and preferences	0.10 (.25)
*CFIR* characteristics	
Development source	0.17 (.24)
Design quality and packaging *Algo design* * Related documents to Algo’s design*	0.10 (.49) 0.18 (.21)
**Context variables** (i.e., Quebec homecare services)	
*PARIHS* characteristics	
Receptive context *Receptivity to the targeted innovation/change*	0.04 (.66)
Culture	0.05 (.57)
Leadership support	0.08 (.36)
Evaluation capabilities	0.01 (.91)
*CFIR* characteristics	
Cosmopolitanism	0.07 (.41)
Peer pressure	0.02 (.85)
External policy and incentives	0.09 (.30)

CFIR = Consolidated Framework for Implementation Research; IKT = integrated knowledge translation.

**Table 2.** Characteristics of Algo's IKT Process in Homecare Services (HCS) According to the PARIHS and CFIR Frameworks

PARIHS dimension *CFIR component*	Statement	Strongly disagree n (%)	Disagree n (%)	Agree n (%)	Strongly agree n (%)
Evidence For the occupational therapists that reach (or not) any level of utilization of Algo (n = 125)					
Patient experiences, needs, and preferences	My workplace (homecare services) collects the view of clients on new practice changes.	24 (19.2)	70 (56.0)	29 (23.2)	2 (1.6)
Characteristics of the targeted evidence-based practice Relative advantage	Algo is (or could be) a useful source of knowledge to meet the needs of equipment selection for our clients’ hygiene care.	5 (4.0)	10 (8.0)	63 (50.4)	47 (37.6)
Clinical experience	In which year did you complete your professional training? How many years have you been working in homecare services?	Years of practice as an occupational therapist $\bar{x}\;(SD)$		Years of practice as an occupational therapist in homecare services $\bar{x}\;(SD)$	
		15.2 (9.2)		9.9 (7.1)	
Research and published guidelines	Which source(s) of knowledge do stakeholders in your workplace value?	Scientific knowledge (i.e., research results)	Clinical knowledge (i.e., professional experience)	Local opinions (e.g., choice of a clinician “leader” in the workplace)	Other
		55 (44.0)	113 (90.4)	66 (52.8)	5 (4.0)
*Intervention characteristics* For the occupational therapists that reach a level of utilization of Algo (n = 50)^3^					
*Development source*	Algo is knowledge designed with the clinical settings of homecare services in Quebec.	0	3 (6.0)	24 (48.0)	23 (46.0)
*Design quality and packaging*	Algo's design (grid) is adequate.	0	1 (2.0)	31 (62.0)	18 (36.0)
	Algo's instructional documents (e.g., reference manual, user guide) are adequate.	0	2 (4.0)	30 (60.0)	18 (36.0)
Context For the occupational therapists that reach (or not) any level of utilization of Algo (n = 125)					
Leadership support	Upon implementation of new knowledge, my role is clearly defined in the work team.	4 (3.2)	37 (29.6)	76 (60.8)	8 (6.4)
Culture	Stakeholders in my workplace, regardless of their respective professions, usually collaborate when implementing a change in practice.	3 (2.4)	24 (19.2)	92 (73.6)	6 (4.8)
Evaluation capabilities	My workplace (homecare services) collects data to assess the new practice changes.	25 (20.0)	54 (43.2)	42 (33.6)	4 (3.2)
Receptivity to the targeted innovation/change	Algo is consistent with the current organizational priorities in my workplace.	10 (8.0)	39 (31.2)	53 (42.4)	23 (18.4)
*Outer setting*					
*Cosmopolitanism*	My workplace collaborates with various external partners (e.g., other homecare services teams, educational institutions, and people in the community who work with us).	2 (1.6)	22 (17.6)	82 (65.6)	19 (15.2)
*External policy and incentives*	In your opinion, do the changes currently occurring in the health care system (sociopolitical level) influence the adoption of new knowledge such as Algo?	3 (2.4)	13 (10.4)	71 (56.8)	38 (30.4)
		Never	Rarely	Occasionally	Frequently
*Peer Pressure*	The decisions of stakeholders working in other homecare services for the adoption of new knowledge such as Algo influence the choices made in my workplace.	6 (4.8)	28 (22.4)	75 (60.0)	16 (12.8)
Facilitation					
For the occupational therapists that reach a level of utilization of Algo (n = 59)		Yes n (%)		No n (%)	
Implementation interventions	Through which communication channel(s) did you hear about Algo? Scientific articles Presentation(s) in congress Algo website *Ergo-Express* During the development of Algo (i.e., *BATH *project) Ongoing training Personal communication (e.g., mail, e-mail) Knowledge broker Word of mouth Other(s)	9 (18.0) 3 (6.0) 18 (36.0) 12 (24.0) 23 (46.0) 1 (2.0) 8 (16.0) 1 (2.0) 30 (60.0) 12 (24.0)		41 (82.0) 47 (94.0) 32 (64.0) 38 (76.0) 27 (54.0) 49 (98.0) 42 (84.0) 49 (98.0) 20 (40.0) 38 (76.0)	
		Strongly disagree n (%)	Disagree n (%)	Agree n (%)	Strongly agree n (%)
Skills and attributes of facilitator	The knowledge broker of my workplace… *… *supports the adoption process of Algo *…*has attributes (e.g., authenticity, accessibility, credibility, respect, reliability) that support the adoption of new knowledge *… *has good skills in areas (e.g., communication [expertise in the application of knowledge], marketing and problem solving) related to the adoption of new knowledge	0 0 0	0 0 0	0 1 (0.8) 1 (0.8)	1 (0.8) 0 0
For the occupational therapists that reach (or not) any level of utilization of Algo (n = 125)					
Other implementation interventions	Among the following strategies, which could help you in the integration of new knowledge such as Algo in your practice? Live on-line training seminar (webinar) Training in your workplace Scientific articles (access to databases) Professional journals (e.g., *Ergo-Express*) Personal communication by mail Personal communication by e-mail	2 (1.6) 3 (2.4) 8 (6.4) 4 (3.2) 17 (13.6) 4 (3.2)	13 (10.4) 11 (8.8) 29 (23.2) 18 (14.4) 36 (28.8) 20 (16.0)	63 (50.4) 50 (40.0) 75 (60.0) 84 (67.2) 65 (52.0) 78 (62.4)	47 (37.6) 61 (48.8) 13 (10.4) 19 (15.2) 7 (5.6) 23 (18.4)

CFIR = Consolidated Framework for Implementation Research; IKT = integrated knowledge translation.

### Characteristics of Algo's IKT Process

**Evidence.** First, relative to the *Evidence* dimension (i.e. Algo), among OTs who knew about the tool (n = 59), a majority of participants (between 88% and 98%; see Table 2) agreed with the facilitative *characteristics of the targeted algorithm* (i.e., design quality). *Clinical experiences* and perceptions about Algo (e.g., content and visual characteristics) were generally positive and adequate for their needs. OTs perceived the design of the tool and the accompanying documents “easy to use and containing a clear rating scale” [OT N.54; Q3]. These findings are further supported by Algo's *relative advantage*, as more than 85% of participants considered it as a useful algorithm.

Concerning the *research and published guidelines’* characteristics, participants tended to value other types of knowledge like clinical knowledge (90%) and local opinions (53%) in greater proportions than scientific knowledge (44%). Algo's IKT development form was well recognized; 94% of participants considered the tool as being developed in partnership with HCS stakeholders. Nevertheless, questions emerged about the “optimal” level of partnership to reach for this collaborative model of knowledge development. Indeed, some participants saw differences between the IKT approach valorized and the one used concretely for Algo. To this end, one participant noted: “There was consultation, but the views of the rehabilitation staff consulted were not necessarily taken into consideration.” [OT N.79; Q4].

Finally, given that *patient preferences* relative to the implementation of new practices appear not to be considered (as indicated by 75% of OTs surveyed), it remains difficult to document feedback from patients having received services with Algo. Thus, according to the *PARIHS* framework, these findings suggest that the facilitative characteristics of the *Evidence* dimension tended to favor Algo's utilization.

**Context.** Regarding the *Context* dimension (i.e., HCS), the influence of the organizational characteristics on Algo's utilization appeared mitigated. On the one hand, some were reported as having the potential to facilitate the progression through the different levels of utilization. For example, the *culture* and *cosmopolitanism* sub-elements were respectively reported by 78% and 81% of participants as being present and positive in HCS (Table 2). A substantial majority of participants also reported being influenced by colleagues (*peer pressure*) to adopt new knowledge occasionally (60%) or frequently (13%).

On the other hand, most other contextual characteristics raised mixed perceptions about Algo's utilization. Even though most OTs (67%) considered that their role during the implementation of knowledge (*leadership support*) is well-defined, OTs reported ambiguities about the role they would like to have during the process. A participant illustrated an aspect of this ambiguity: “Although this new knowledge is related to OT, managers do not tend to consult OTs to make changes to their functioning.” [OT N.124; Q11]. The reported lack of mechanisms for following implemented practices also led to question the *evaluation capabilities* of HCS to document benefits and difficulties related to Algo's utilization. In support of this finding, a participant said: “Little time is now given to evaluate practices; organizational changes take center stage unfortunately.” [OT N.107; Q07].

Although *external policies and incentives* (i.e., ongoing restructuration of Quebec health care system) are perceived to influence the implementation of new practices by most participants in HCS (87%), its impact is however viewed differently. Some OTs perceived it as a barrier: “But we are all out of breath by the speed at which changes in practices currently occur. […] Impacts on our sense of competence and efficiency at work.” [OT N.58; Q10]. Others see the reorganization of the health care system as an opportunity: “Currently, we are, like in all the *CI[U]SSS*, in the process of harmonization. This is an opportunity to keep ourselves up to date in the targeted areas.” [OT N.115; Q07]. Along the same line of thought, even if the *OEQ* published guidelines on the collaboration between OTs and nonOTs, the absence of policies and procedures to this end acts as a potential barrier, as exemplified by a participant who said: “If there were policies and procedures to frame Algo, it would be more obvious.” [OT N.114; Q04]. Thus, according to the *PARIHS* framework, the ambiguous *Context* characteristics tended to slow the utilization of Algo.

**Facilitation.** For the *Facilitation* dimension (i.e., strategies deployed to sustain Algo's utilization), strategies used by OTs who know the algorithm were mainly: (1) word of mouth (60%), (2) the research project related to the co-development of Algo (46%), and (3) the website presenting the algorithm (36%). The use of these strategies for Algo's IKT process tended to differ from those valued in their professional practice. Indeed, the participants reported prioritizing the: (1) webinar (training on the web; [88%]), (2) training in the workplace (89%), (3) professional journals (82%), and (4) communication by e-mail (81%) for integrating new knowledge (Table 2).

**Figure 1.**
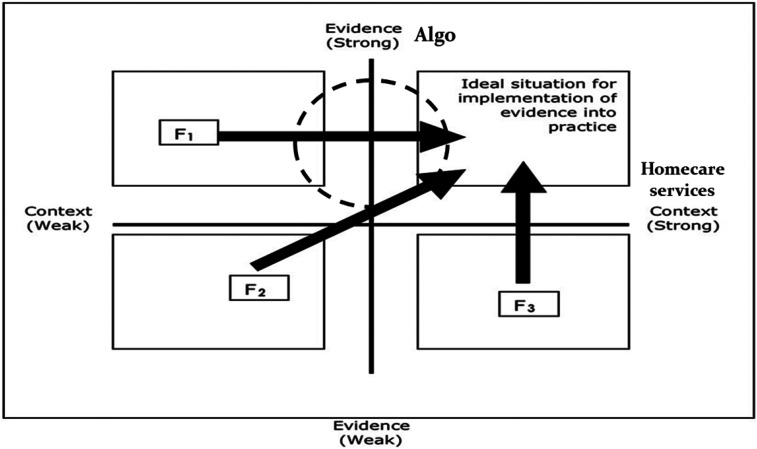
Algo's integrated knowledge translation (IKT) process in homecare services (HCS) according to the PARIHS framework (Kitson et al., 2008).

While several of Algo's characteristics (*Evidence*) seem to have contributed to its dissemination and the initiation of its use by OTs, HCS statements (*Context*) tend to indicate an unfavorable local and organizational climate to Algo's implementation. Globally, these characteristics allow to situate the IKT process of Algo halfway between the first quadrant (F1) and the ideal situation for implementation of evidence into practice (Figure 1). Facilitation strategies need therefore to consider additional contextual characteristics to support Algo's utilization in HCS.

### Discussion

The purpose of this study was to identify the level of utilization of Algo 2 years after its diffusion, and the characteristics associated with a higher level of its utilization in HCS. These results indicate that in 2015, the majority of OTs working in HCS that have been reached in this study did not use Algo in an instrumental manner in their professional practice. However, almost 40% of targeted OTs in this study and aware of Algo have been able to attain different levels of utilization. This proportion is encouraging given the average 17-year gap documented in the KT literature between the production and utilization of knowledge (Morris et al., 2011).

Several *Evidence* characteristics of Algo's IKT process appeared to facilitate its utilization among OTs in HCS. For example, the simple and easy-to-understand design of Algo (and its related documents) was reported as being an enabling feature for moving through the initial levels of utilization, once the reception stage is passed (i.e., cognition, reference). The co-production mode used for Algo's development promoted the consideration of OTs’ preferences and needs as well as the published guidelines on bathing equipment (Guay et al., 2012, 2013, 2014). Despite this positive element, the optimal level of involvement of knowledge users in the co-creation process could be further defined. According to some participants, the IKT criteria applied for the development of Algo seemed insufficient to prompt their full improvement. From this perspective, the mixed receptivity regarding the procedures related to the development of knowledge with IKT approaches could explain the choice of some professionals to stop the utilization process through intermediate stages. However, the positive perceptions of Algo's supervisors (i.e., OTs) regarding the scientific-based development of Algo nevertheless remain an important facilitator to its utilization. Indeed, the value attributed to evidence-based knowledge is an important facilitator recognized in OT for the implementation process (Swedlove & Etcheverry, 2012).

These interpretations about the *Evidence* characteristics of Algo IKT's process can be corroborated with the significant statistical associations found and the regression model developed with *Evidence* characteristics. Even though the association between the value of scientific knowledge and the achievement of a higher level of utilization of Algo remains moderate, it suggests that the evidence-based development of the algorithm is an important characteristic to be considered for its utilization among professionals who tend to prioritize scientific knowledge. Indeed, Algo is the only evidence-based algorithm available among the in-house “tools” used in Quebec HCS (Guay et al., 2019). The relative advantage of Algo, the second statistically suggestive characteristic associated with an advanced level of utilization and integrated into the regression model presented above, also appears as an important variable to consider regarding the promotion of its IKT process. In this case, even if the regression model explained only 21.5% of the variance, these results suggest that the *Evidence* dimension is taken into consideration by OTs when deciding to reach higher levels of utilization. The relative advantage is a well-recognized facilitating characteristic in the KT field (Hunter et al., 2017; Lindholm et al., 2019) and the results of this study suggest that a high level of agreement about Algo's relative advantage may influence the behavior of OTs who are progressing through the levels of utilization. A cross-sectional study (Peels et al., 2014) supports this finding, as the characteristics and the relative advantage of health behavior interventions were found to be associated with the intention of implementing the change (which corresponds to the initial and intermediate levels of utilization of Algo). The characteristics of Algo as an evidence-based algorithm should therefore continue to be valued and increasingly promoted in the next steps of facilitation.

Features of Quebec HCS (*Context*) are among the main barriers to Algo's utilization, according to the OTs included in this study. Indeed, although the organizational culture represents a potential facilitator to Algo's IKT process, the increase of skill mix and in-house “tools” use in Quebec HCS documented during the development of the tool (Guay et al., 2019) has evolved in a context described by the absence of clear evaluation mechanisms following the implementation of a change. Even though Algo is perceived as a tool that aligns with organizational priorities (e.g., accelerating services offer to the population), the lack of evaluation abilities regarding the implementation of new practices and the scarcity of policies and procedures to manage the use of skill mix in interprofessional teams seem to hamper the progression of Algo through intermediate and advanced stages of utilization. Leadership is well-documented as an influential characteristic for implementing evidence-based practices (Aarons et al., 2014; Li et al., 2018; Powell et al., 2017). Given the ambiguity identified toward the leadership support received by OTs from the administrative stakeholders to implement change, it appears important to focus on this characteristic since it tends to hinder the IKT processes of clinical tools such as Algo. Indeed, even if the administrative stakeholders appear collaborative in the planning of such implementation processes, the decision-making power of organizational instances seems to weaken the substitution process, involving both the deimplementation of in-house “tools”, as well as the implementation of validated knowledge (as Algo). This functioning evolves in a context where skill mix tend to be more used in homecare OT (Guay et al., 2019), while the mechanisms to rigorously control its recourse seem little managed.

The contextual characteristics identified in this study should be considered in light of the health system reform initiated in 2015 in Quebec that led to a complex reconfiguration of structures (Assemblée nationale du Québec, 2015). It is possible that the resulting contextual instability has contributed to the difficulties previously identified. Globally, characteristics related to the *Context* dimension tend to slow the progression of Algo through the intermediate and advanced levels of utilization. Although contextual characteristics are known to influence the outcome of an implementation process (Grimshaw et al., 2012; Li et al., 2018), associations between the *Context* characteristics and the level of utilization of Algo have not been identified in this study. These results could be partially explained by the fact that the characteristics were documented from an individual perspective. The absence of data from stakeholders involved in other contextual levels (e.g., HCS managers) led to portray partly this dimension of the *PARIHS* framework. This finding is in line with the question raised by Schultz and Kitson (2010) regarding the most suitable unit of measurement for context in implementation studies. More detailed measures of individual and contextual factors may therefore be necessary to understand the interrelation between these concepts as well as identifying the impact of contextual levels on the implementation process. While Algo's IKT process aligns, for the most part, with *PARIHS*'s recommendations on the successful implementation of evidence (Figure 1), only approximately half of the OTs (47.2%) reached one of the seven levels of utilization of Algo. In this perspective, this representation of the process might be further nuanced by deepening the contextual levels and better situating visually the evolution of the process.

Regarding the *Facilitation* strategies used for promoting the utilization of Algo, word of mouth was one of the main strategies used by OTs to know about Algo. Indeed, the scientific literature showed that health professionals rely considerably on peers for learning about knowledge relevant to their practice (Ketelaar et al., 2008). Although the knowledge broker constitutes a type of facilitator discussed in the KT literature (Bornbaum et al., 2015), it may not be as prevalent in HCS as only one OT reported having access to this resource. Contrary to the *PARIHS* conceptualization, it should be noted that facilitation strategies for Algo's IKT process have not been developed following an analysis of *Evidence* and *Context* characteristics. However, through the IKT form used to develop Algo, different facilitative characteristics have been identified through an operational knowledge translation and exchange framework (Guay et al., 2019). Indeed, some elements related to the end users’ characteristics have been effectively documented and led to the definition of a facilitative *Evidence* dimension for the IKT process of Algo. In this study, considering that more than half of the participants were not reached with the initial strategies targeting end users of Algo for its implementation, future facilitation efforts should consider additional characteristics related to the individuals. An adjustment of the strategies should also be considered regarding the *Context* characteristics (e.g., organizational priorities of HCS’ managers).

### Limitations

The main limitation of this study lies in the impossibility to verify the representativeness of the sample, given that data on OTs working specifically in Quebec HCS is unavailable. Indeed, information about only two sociodemographic characteristics (i.e., gender and administrative region of professional practice) of the general population of Quebec OTs was available. The participation rate (16%) is however similar to those observed in KT studies using this method among this population (Thomas & Law, 2014). The generalizability of the study's findings could be questioned since we can assume that OTs reached by the electronic survey were actually interested by Algo. This limitation has been minimized by the fact that OTs who were not aware of Algo could nevertheless answer a substantial number of questions and statements. Further exploration of OTs’ values would also be relevant to detail their consideration of Algo comparatively to the in-house “tools” used. Although the opinion gathered from OTs, as supervisors of Algo users, is at the core of studying this IKT process, the point of view of other end users (e.g., nonOTs) and HCS managers would have provided a more comprehensive scheme. For the qualitative component, the limit related to the coding of utterances (i.e., only performed by a member of the research team) has been counterbalanced by the use of a deductive approach rooted in recognized KT conceptualizations. Finally, since the study may constitute a facilitation strategy itself, the possibility that the research project influences the subsequent process should be noted. However, the documentation of this research as a facilitation strategy in future studies will allow to consider its subsequent impact.

### Strengths

This study relied on strong and extensive theoretical frameworks to identify the diverse characteristics describing the implementation process of a clinical algorithm developed with an IKT approach. Indeed, the development of the questionnaire as well as the collection and analysis of the data were based on well-recognized conceptualizations (i.e., *PARIHS* and *CFIR*) and rigorous pretest procedures. The sample size (n = 125) was also sufficient to perform supplementary statistical analyses (e.g., logistic regression) to deepen the analysis of the characteristics related to Algo's utilization.

## Conclusion

In OT, a clinical algorithm (Algo) developed within an IKT approach is currently being disseminated and used among different HCS. A KT portrait of characteristics related to its utilization, according to the *Evidence* and *Context* dimensions, allowed to target its associated variables as well as some of the facilitating orientations that could be taken to continue its implementation. Now that Algo has been welcomed by many OTs, efforts aimed at the different contextual levels will be necessary to reach advanced levels of utilization. An adjustment to the facilitation strategies will have to be considered to target other stakeholders involved in the process. This study initiated the theoretical and clinical analysis of the implementation implications for tools developed and disseminated within an IKT approach in homecare OT.

## Key Messages

- Algo, a clinical algorithm supporting skill mix in homecare services (HCS) to select bathing equipment for patients living at home with difficulties accomplishing hygiene care, is an integrated knowledge translation (IKT)-based decision tool deployed since 2013 in occupational therapy (OT).
- As currently defined, the IKT approach used in the development of Algo facilitated the initiation of its application among OTs and nonOTs (e.g., home health aides), mainly because of the *Evidence* (i.e., Algo) characteristics considered through the perspective of knowledge end users.
- Given that unfavorable *Context* (HCS) characteristics tend to explain several difficulties encountered for Algo's implementation, ulterior *Facilitation* and IKT initiatives should embrace additional contextual characteristics related both to the potential knowledge users and context.

## Notes

1. **p* ≤ .05.

2. ***p* ≤ .005.

3. Missing data about the intervention characteristics were identified for nine participants.
